# Association of Vitamin D Deficiency and Degree of Coronary Artery Disease in Cardiac Patients with Type 2 Diabetes

**DOI:** 10.1155/2017/3929075

**Published:** 2017-11-02

**Authors:** Ewelina A. Dziedzic, Jakub S. Gąsior, Mariusz Pawłowski, Marek Dąbrowski

**Affiliations:** ^1^Cardiology Clinic of Physiotherapy Division of the 2nd Faculty of Medicine, Medical University of Warsaw, Warsaw, Poland; ^2^Department of Cardiology, Bielański Hospital, Warsaw, Poland; ^3^Faculty of Health Sciences and Physical Education, Kazimierz Pulaski University of Technology and Humanities, Radom, Poland

## Abstract

Several modifiable factors may influence cardiac function in diabetic patients. The aim of the study was to evaluate the influence of vitamin D level on the stage of coronary atherosclerosis in cardiac patients diagnosed with type 2 diabetes. The study was performed in 337 consecutive patients undergoing coronarography. The stage of atherosclerosis was evaluated using Coronary Artery Surgery Study Score. The plasma 25(OH)D concentration was determined by an electrochemiluminescence method. Patients without significant lesions in coronary arteries presented the highest 25(OH)D level, significantly higher than patients with one-, two-, and three-vessel coronary artery disease (CAD) (*p* < 0.01). Significantly lower level of the 25(OH)D was observed in patients hospitalized due to acute coronary syndrome (ACS) in comparison to patients hospitalized due to stable CAD (*p* < 0.001). Lower 25(OH)D levels were observed in patients with the history of myocardial infarction (MI) in comparison to patients without previous MI (*p* < 0.001). In cardiac patients with diabetes, the higher number of stenotic coronary arteries is associated with lower values of the 25(OH)D. A group of male cardiac patients with diabetes with significant stenosis in three coronary arteries, hospitalized due to acute coronary syndrome, with a history of previous MI and hyperlipidemia presented the lowest vitamin D level.

## 1. Introduction

Type 2 diabetes mellitus (T2DM) is related to many complications including macroangiopathy, and it is known to be one of the main risk factors for coronary disease [1, 2]. The overall mortality in patients with T2DM doubled, while the mortality related to cardiovascular diseases tripled in comparison to that of nondiabetic patients over the past few years [3]. It has been estimated that by the end of 2030 T2DM will have been diagnosed in 552 million people around the world which shows the growing gravity of this health problem [4].

The pathogenesis of diabetes is multifactorial, and the process of glycemic control is very complex [5, 6]. Actually, in order to improve the treatment effectiveness as well as diabetes complication prevention, new potentially modifiable factors influencing effective glycemic control need to be discovered. As such, the role of vitamin D deficiency has been recently suggested [7–13]. Vitamin D, via regulation of calcium, influences various mechanisms associated with the pathophysiology of T2DM, including pancreatic *β* cell dysfunction and impaired insulin action [14–16]. Based on the longitudinal observational studies, it was suggested that optimizing vitamin D level could reduce insulin resistance [17], improve glycemic control [8, 18], and consequently reduce the risk of T2DM [10, 19]. However, the results from underpowered trials and post hoc analyses of larger trials on the effect of vitamin D supplementation on glycemic outcomes have been inconsistent [14, 20–25].

With the discovery of vitamin D receptors in many body tissues, new links between diabetes and cardiovascular diseases have been studied [26]. It has been shown that vitamin D affects endocrine and cardiovascular systems via its nuclear receptor (vitamin D receptor (VDR)) [27]. The expression of VDR was observed in pancreatic *β* cells, immune system cells, vascular endothelium, vascular smooth muscle, and cardiomyocytes, which are capable of autocrine synthesis of the vitamin D active metabolite (1,25-dihydro-xyvitamin D) [28]. Seasonal changes of glycemic control efficacy in patients with diabetes may be explained by the role of vitamin D in the pathogenesis of glucose metabolism disorders. In the autumn-winter season, glycemic control is compromised, which might be related to vitamin D deficiency as its main source is skin production after exposure to sunlight solar radiation [29]. Vitamin D deficiency is considered a so-called “nonclassical” cardiovascular risk factor [30]. It was shown that low 25(OH)D level was associated with prevalent coronary artery disease (CAD) independent of other recognized cardiovascular risk factors [31]. As it was shown in National Health and Nutrition Examination Survey (NHANES) concerning adults from the United States, patients with low level of 25(OH)D (less than 21 ng/ml) have an increased risk of, for example, hypertension, diabetes, and obesity [32, 33]. Nevertheless, we have presented recently that in the whole group of subjects, that is, a numerous heterogeneous Polish cardiac patient population including subjects with, for example, hypertension, diabetes, and/or hyperlipidemia, there was no significant association between the level of the 25(OH)D and the stage of coronary atherosclerosis [34]. An additional independent analysis revealed that patients with diabetes presented lower values of the 25(OH)D than nondiabetic and that the association of the 25(OH)D and the stage of coronary atherosclerosis may depend on the diabetic status (data not shown). In nondiabetic cardiac patients, there was no significant association between the vitamin D level and the stage of coronary atherosclerosis (results of analysis of variance), whereas, in the combined group of patients with one to three vessel atherosclerosis, significantly lower 25(OH)D level was presented compared to patients without significant lesions in the coronary arteries. Moreover, lower 25(OH)D level was shown in patients hospitalized due to acute coronary syndrome compared to patients diagnosed with stable CAD (data not yet published).

The presented study focused on the association between the 25(OH)D level and the severity of coronary artery atherosclerosis in cardiac patients with type 2 diabetes.

## 2. Methods

### 2.1. Population

This study is part of a wider research project aimed at investigating the association between the vitamin D level and severity of coronary artery atherosclerosis in Polish cardiac patients. The previous analysis concerned about six hundred patients examined using coronary angiography that we included into the general analysis. Detailed characteristics of the overall study sample are presented elsewhere [34]. Additional analysis aimed to find determinants of the degree of coronary atherosclerosis showed that one of the independent significant determinant was diabetic status (two groups of patients: without diabetes and with type 2 diabetes). Results for nondiabetic cardiac patients were not yet published. Since then, we have continuously examined new patients. The results obtained for diabetic patients regarding the association of vitamin D deficiency and coronary artery status are shown in the presented manuscript. The study comprises data of patients hospitalized in Cardiology Department who underwent cardiac catheterization from 2013 to 2017. Coronary angiography, performed due to suspected coronary artery disease, was the sole inclusion criteria for the study. In each patient, serum phosphate, calcium, and parathyroid hormone levels were measured. Participants with disturbances of calcium and phosphate metabolism were excluded. Exclusion criteria were chronic kidney disease (stage III-V) due to accompanying disorders of calcium and phosphate, diagnosed cancer (paraneoplastic syndromes and associated disorders of calcium phosphate), elevated markers of inflammation (total white blood cell count > 10,000 cells/*μ*l or C-reactive protein level > 5 mg/l) or fever, and medication or supplementation containing vitamin D or calcium. The enrolled patients were only Polish citizens, so it may be presumed that in terms of eating habits and the length of exposure to sunlight they constitute an almost homogeneous group.

All patients with diabetes were also treated with statins (atorwa or rosuvastatin). Written informed consent was received from all the patients participating in the study. The research was approved by the University Bioethical Committee and followed the rules and principles of the Helsinki Declaration.

### 2.2. Measurements

Data were collected using an interview questionnaire (age, ethnicity, smoking habits, presence or absence of previous myocardial infarction (MI), coronary artery disease (CAD) status defined by clinical history of acute coronary syndrome (ACS) or stable coronary artery disease (stable CAD), and measurements of diabetes status, body mass index (BMI), level of total cholesterol (TC) and/or triglyceride (TG) to define the presence or absence of hyperlipidemia, systolic and diastolic blood pressure to determine the presence or absence of hypertension, total 25-OH vitamin D in participant serum and plasma, and coronary angiography to assess the degree of coronary atherosclerosis).

Diabetes was diagnosed when a fasting plasma glucose level was ≥126 mg/dl (7.0 mmol/l) or a casual plasma glucose measured at any time was >200 mg/dl (11.1 mmol/l) [35] or the patient was treated for type 2 diabetes before hospitalization.

Standing height and weight were measured during the physical examination and were used to calculate BMI (kg/m^2^). Hyperlipidemia was defined when the TC > 200 mg/dl and/or TG > 150 mg/dl. The levels of TC and TG were measured using the enzymatic method. Hypertension was defined as a systolic blood pressure ≥ 140 mm Hg or diastolic blood pressure ≥ 90 mm Hg.

Elecsys Vitamin D Total assay (Roche Diagnostics, Mannheim, Germany) was used for the quantitative determination of total 25-OH vitamin D (the major storage form of vitamin D in the body) in participants' serum and plasma. This competitive electrochemiluminescence assay evaluates the levels of both 25(OH)D2 and 25(OH)D3. The sensitivity of this assay was 4.01 ng/ml, and the coefficient of variation was 18.5%. Liquid chromatography-tandem mass spectrometry (LC-MS/MS), currently considered as the gold standard to determine the level of 25(OH)D, allows for the independent determination of 25(OH)D3 and 25(OH)D2 [36]. Nevertheless, due to the relatively high equipment costs, its widespread clinical use is limited [37]. Use of less expensive methods to determine the level of both total metabolites of vitamin D is warranted because the biological effects of both metabolites of vitamin D are similar. It was indicated that Elecsys “Vitamin D Total” Assay is comparable to LC-MS/MS and appropriate for clinical use [38–40]. Level of 25(OH)D < 10 ng/ml is considered as severe deficiency, ≥10 to <20 ng/ml as moderate deficiency, ≥20 to <30 ng/ml as mild deficiency, and ≥30 ng/ml as optimal [33]. Ultraviolet B radiation in sunshine associated with the season of the year contributes to the production of vitamin D in the skin [41–43]. Participants in our study were examined throughout the whole year on what may influence their 25(OH)D level. Examination data corresponds to the season of blood draw, which was reported by NHANES as winter months (November to April) and summer months (May to October) [44]. In all patients, determination of 25(OH)D was performed before coronary angiography.

The coronary angiography was performed using standard diagnostic catheters through radial or femoral artery access. The Coronary Artery Surgery Study Score (CASSS) was used to assess the degree of coronary atherosclerosis [45]. Stenosis > 70% in any of the large epicardial coronary arteries (anterior descending branch, circumflex branch, and right coronary artery) was scored at 1 point. Stenosis ≥ 50% of the left main coronary artery was scored at 2 points and considered 2-vessel disease. The score was calculated as the sum of all points. The score may illustrate 1-, 2-, or 3-vessel CAD [45].

### 2.3. Statistical Analysis

The Wilk-Shapiro test was used for evaluating the normal distribution of data. The Poisson regression analysis was used to assess the relationship between the degree of coronary atherosclerosis (CASSS) and selected variables: level of vitamin D (25(OH)D), age, sex, BMI, smoking habits, hypertension, hyperlipidemia, coronary artery disease (CAD) status, previous myocardial infarction (MI), and examination data. Logistic regression was used to assess the association between 25(OH)D level and other selected covariates with CAD status. To determine the influence of age, sex, BMI, smoking, hypertension, hyperlipidemia, previous MI, and/or examination data on 25(OH)D level, the multivariable regression analysis was carried out. Continuous variables were compared between the three or more groups using analysis of variance (ANOVA; followed by Tukey unequal N HSD post hoc tests) or the Kruskal-Wallis test (followed by post hoc Dunn's multiple comparison test) depending on the data distribution. To compare the results of continuous variables between the two groups, Mann–Whitney *U* test was used. Pearson's chi-squared test was used to determine eventual differences between prevalence in selected groups. Statistical analyses were performed with a significance level of 5% (*p* value < 0.05). The statistical analysis was carried out with STATISTICA 12.5 software. The figure was created using GraphPad Prism 5.0 (GraphPad Software Inc., La Jolla, CA, USA).

## 3. Results

### 3.1. Participants' Characteristics

The study group consisted of 337 patients (218♂ and 119♀) with mean (±SD) age and BMI: 68.1 (±10.3) years and 30.3 (±5.3) kg/m^2^. Active smoking was declared by 84 patients (24.9%). There were 224 nonsmokers (66.5%) and 29 of patients who declared that they are ex-smokers (8.6%). Hyperlipidemia was observed in about half of the patients (174 patients—51.6%) whereas hypertension in most of them (312 patients—92.6%). ACS was a cause of hospitalization in 136 (40.4%) and stable CAD in 201 patients (59.6%). Previous heart attack was reported by 45.1% of the patients. Examination from May to September was performed in 70 patients (20.8%), from October to May in 267 patients (79.2%).

The median of 25(OH)D in the whole group was 14.5 ng/ml (4.0–43.9 ng/ml). The level of 25(OH)D equal or greater than 30 ng/ml (optimal level) was observed in 16 patients (4.8%), whereas the level equal or below 10 ng/ml (severe deficiency) was noticed in 78 patients (23.2%). Concentration equal or greater than 10 ng/ml and lower than 20 ng/ml and equal or greater than 20 ng/ml and lower than 30 ng/ml was observed in 168 (49.9%) and 75 patients (22.3%), respectively.

Insignificant changes in the coronary arteries (CASSS 0) were found in 66 patients (19.6%). Single-vessel disease (CASSS 1) was discovered in 79 patients (23.4%), two-vessel (CASSS 2) in 98 (29.1%), and triple-vessel (CASSS 3) in 94 patients (27.9%).

### 3.2. Association of Vitamin D Level and Other Factors with CASSS

**Figure 1:**
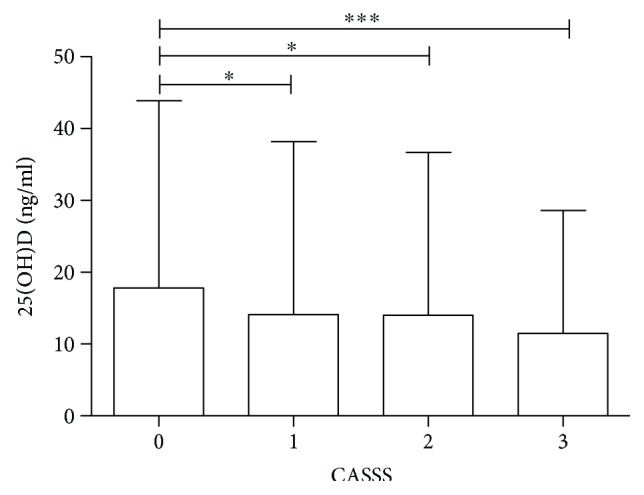
The level of 25(OH)D with respect to the CASSS subgroup. ^∗^*p* < 0.05; ^∗∗∗^*p* < 0.001.

Detailed characteristics of patients in four subgroups according to CASSS are shown in Table 1. There were significant differences in the 25(OH)D level between the groups with respect to the degree of coronary atherosclerosis (*p* < 0.001). Patients with CASSS 0 presented significantly higher median 25(OH)D value than patients in all other CASSS subgroups, that is, from 1 to 3 (Figure 1). The lowest and the highest number of patients with level of 25(OH)D below 10 ng/ml was observed in CASSS 0 and CASSS 3 subgroups, respectively. The results showed a statistical significance in the prevalence of patients divided according to sex, CAD status, and previous MI among CASSS subgroups. There were more males than females in CASSS subgroups from 1 to 3. The number of male and female patients in CASSS 0 subgroup was equal. Independently, the proportions of patients with stable CAD to ACS and without previous MI to the presence of previous MI were the highest in CASSS 0 subgroup. More patients with a history of previous MI than without previous MI were noticed in CASSS 2 and 3 subgroups. There were no significant differences in age and BMI between CASSS subgroups. There was no statistical significance in the prevalence of hypertension and hyperlipidemia among different stages of coronary atherosclerosis. Smoking status was similar in all CASSS subgroups. Also, in all subgroups, the majority of patients were examined between November and April. Due to significant differences in the 25(OH)D level among patients with CASSS 0 and CASSS from 1 to 3, the overall group was divided into two subgroups: patients without significant stenosis in any of the large epicardial coronary arteries (CASSS 0) and patients with significant stenosis in 1, 2, and/or 3 coronary arteries (CASSS 1–3) (Table 2). From selected variables, sex, 25(OH)D, and previous MI proved to be significant determinants of CASSS (Table 3).

### 3.3. Association of Vitamin D Level with Clinical Presentation

Patients with ACS presented significantly lower values of 25(OH)D than patients with stable CAD (Table 4). There was a statistical significance in the prevalence of patients in the subgroups of CASSS. The proportion of the patients with stable CAD to ACS was the highest in CASSS 0 subgroup and the lowest in CASSS 3 subgroup. There was statistical significance in the prevalence of patients divided according to smoking habits, previous MI, and examination data among two groups of CAD status. In the logistic regression analysis, from selected determinants, a history of previous MI and examination data were statistically significant (Table 5).

### 3.4. Association of Different Factors with Vitamin D Levels

The presence of hyperlipidemia, history of previous MI, and examination between November and April were significantly associated with lower values of 25(OH)D in cardiac patients with diabetes (Table 6).

## 4. Discussion

In this study, the association between 25(OH)D level and the severity of coronary atherosclerosis was clearly observed; that is, we showed that diabetic cardiac patients with CASSS 0 presented a significantly higher vitamin D level in comparison to patients with one-, two-, and three-vessel coronary atherosclerosis (CASSS 1, 2, and 3). Patients with CASSS 3 presented the lowest values of 25(OH)D. The history of previous MI was significantly and independently associated with both higher number of vessels with significant stenosis and a lower vitamin D level. Similarly to the group of nondiabetic patients, a significantly lower 25(OH)D level was also noticed in diabetic subjects hospitalized due to ACS compared to patients diagnosed with stable CAD. However, diabetic patients hospitalized due to ACS presented lower value of 25(OH)D than those without diabetes.

Multiple studies suggested that hypovitaminosis D is associated with an increased risk of cardiovascular diseases and diabetes [30, 46–49]. CAD is the most common complication of diabetes and the leading cause of death in this group of patients [50–52]. It is estimated that CAD accounts for 50% deaths in diabetic patients and the coexistence of diabetes and coronary heart disease causes a 2-3-fold increase in the risk of death in men and 3–5-fold increase in women [53]. Considering the documented, widespread vitamin D deficiency in Polish population [54], the aim of our research was to evaluate the relationship between a vitamin D level and the stage of coronary disease in patients at risk of cardiovascular death.

In our previous study performed in over 600 Polish patients including diabetic and nondiabetic individuals, low 25(OH)D values and no relationship between a vitamin D level and the stage of coronary atherosclerosis were shown. The mean value of vitamin D in the overall population was 15.9 ng/ml [34]. A detailed analysis has revealed that 25(OH)D level was significantly lower in diabetic (14.5 ng/ml) than nondiabetic (15.0 ng/ml) patients. The values presented are lower than those in the epidemiological studies performed, for example, in the United States [32, 55–58]. In the Atherosclerosis Risk in Communities (ARIC), the overall study population median 25(OH)D concentration was 23.9 ng/ml [57]. In the ARIC study, in a selected group of subjects, the value of 25(OH)D concentration < 17.2 ng/ml was associated with a higher stroke risk [57] and an increased risk of incident coronary heart disease [58]. The lower values of 25(OH)D concentration in our study in comparison to other studies may be associated with the method of vitamin D measurement. Elecsys Vitamin D Total assay was used in our patients to determine the total 25-OH vitamin D level. It should be mentioned here that in 2011 Connell et al. concluded that the Roche Elecsys Vitamin D3 assay presented unacceptable performance and, thus, underestimated vitamin D level [59]. Notwithstanding, the values resulting from our study are consistent with those from the observational epidemiological data on the vitamin D status in Polish population despite the different method of vitamin D measurement [54]. Pludowski et al. showed massive vitamin D deficiency in Poland [54]. In about six thousand healthy people from more than twenty Polish towns, the mean value of the 25(OH)D was 18.0 ng/ml. The optimal values (i.e., above 30 ng/ml) were observed in less than ten percent of the population [54]. Similar results were obtained by another Polish team; more than 80% of adults living only in the north of Poland demonstrated the 25(OH)D level below 20 ng/ml [60]. To assess the vitamin D level, the “DiaSorin LIAISON® 25OH Vitamin D TOTAL assay” was used by both research groups [54, 60].

The low level of vitamin D in cardiac diabetic patients may be one of many factors leading to the destabilization of atherosclerotic plaque and, in consequence, to the local formation of blood clot and subsequent myocardial infarction. In addition to a thin connective tissue layer on the surface of the plaque, large lipid nucleus, excessive inflammatory response, and increased neovascularization play a key role in the process of the destabilization of atherosclerotic plaque [61–63]. The study showed that besides the well-described anti-inflammatory effects, an adequate level of vitamin D reduces the activity of metalloproteinase enzymes responsible for the degradation of extracellular matrix components and the destruction of fibrous envelope [64]. Moreover, vitamin D inhibits the vascular endothelial growth factor and stimulates epithelial cell apoptosis, which in consequence inhibits neovascularization within existing atherosclerotic plaques that leads to their stabilization and may prevent myocardial infarction [65]. Vitamin D exerts anticoagulant properties through mechanisms such as downregulation of procoagulant tissue factor and upregulation of thrombomodulin [65, 66] and the inhibition of various adhesion molecule expressions, thus preventing platelet activation and decreasing fibrinolysis and thrombosis [67].

Our results presenting the relationship between low levels of 25(OH)D with MI in patients with significant stenosis in coronary arteries and type 2 diabetes are consistent with other published data [68]. Although the definition of vitamin D deficiency differs in various studies, it was shown that a low level of 25(OH)D in diabetic patients is related to an increased number of multivessel lesions [68], a higher risk of cardiac nosocomial death [69], a higher risk of asymptomatic CAD [70], a poor glycemic control, and a higher serum level of inflammatory markers [71]. Gondim et al. examined 166 patients with type 2 diabetes diagnosed with ST segment elevation myocardial infarction (STEMI) and observed a higher percentage of vitamin D deficiency (defined as a level below 20 ng/ml) compared to patients without STEMI. In addition, a higher percentage of multivessel lesions was observed in diabetic patients with vitamin D deficiency compared to nondiabetic patients [68]. Other authors have shown that severe vitamin D deficiency (defined as the 25(OH)D level below 10 ng/ml) is associated with a higher risk of cardiovascular nosocomial death in patients with type 2 diabetes hospitalized due to acute coronary syndrome. The risk of nosocomial pneumonia and death in this group of patients was 24%, whereas, in comparison, the risk in patients with higher levels of 25(OH)D was only 5% [69]. In a group of two hundred patients with type 2 diabetes with urinary albumin excretion without symptoms of CAD, Joergensen et al. have demonstrated that serious vitamin D deficiency (<5 ng/ml) is related to an increased risk of asymptomatic coronary disease [70]. O'Hartaigh et al. have studied a group of diabetic patients with a high risk of cardiovascular events and suggested that those with lower levels of 25(OH)D had worse glycemic control and higher levels of inflammatory markers in serum [71].

The results discussed above together with our findings suggest that there is an association between a low level of vitamin D and a stage of coronary artery disease expressed as a number of stenotic coronary arteries in cardiac patients with T2DM, particularly in patients hospitalized due to ACS with a history of previous MI and hyperlipidemia. Taking into account factors associated with the number of coronary arteries with significant stenosis and a determined level of 25(OH)D, we identified a group of cardiac patients with diabetes who may be mostly predisposed to vitamin D supplementation. Such a group consists of male patients with significant stenosis in three coronary arteries, hospitalized due to ACS, with a history of previous MI and hyperlipidemia. The 25(OH)D level in the group was below 10.6 ng/ml. If a patient with mentioned characteristics was examined to assess vitamin D level from November to April, clinicians and researchers might expect nominally lower value of vitamin D level, even less than 10 ng/ml, that is, about 8-9 ng/ml. However, our study is observational; thus, it cannot determine the impact of intervention such as vitamin D supplementation and/or exposure to summer sun on, for example, the degree of coronary atherosclerosis in diabetic cardiac patients.

In the United States, a low level of vitamin D is linked to important risk factors of leading causes of death [72]. Some authors indicated that clinicians should be aware of this connection and offer, for example, a dietary intervention to increase vitamin D level, especially in minority groups [72–78]. Professional guidance on vitamin D supplementation was suggested [79, 80]. Actually, it seems that vitamin D has beneficial effects only in subjects at risk of diabetes [14, 23, 81, 82]. Authors of recent systematic review and meta-analysis of vitamin D treatment in adults with type 2 diabetes found a modest reduction of hemoglobin A1C and no difference in fasting blood glucose [83]. Wu et al., also based on systematic review and meta-analysis, concluded that vitamin D supplementation could be effective at improving glycemic control, however, in selected group of diabetic patients, that is, in those with 25(OH)D deficiency at baseline or nonobese (with BMI < 30 kg/m^2^) [84].

Considering the fact that diabetic patients are at high risk of cardiovascular disorders and cardiovascular diseases remain the main cause of death in this group, there is an urgent need to perform further studies elucidating the role of vitamin D in the development of cardiovascular disease and its complications in cardiac patients with glucose metabolism disorders. Future randomized studies to assess the effects of vitamin D supplementation will show if such intervention could be beneficial for cardiac patients with diabetes.

### 4.1. Limitations

Several issues regarding study limitations should be taken into consideration. The Elecsys Vitamin D Total assay was used; thus, the 25(OH)D level can be underestimated [59]. The study was carried out retrospectively without the assessment of the diabetes treatment efficacy (the level of glycated hemoglobin has not been measured). The study was performed in a group of patients from central Poland, and most subjects lived in urban areas. The study should be extended to the inhabitants of other regions of Poland, so it would be possible to translate the obtained results into the entire Polish population. Another limitation of the study is the classification of atherosclerosis stage based on coronary angiography and CASSS classification. The classification does not consider the occurrence of calcification in coronary arteries that stabilize atherosclerotic plaques. All participants of the study were treated with statins; however, the data analyzed in the study does not include the information on the dose or treatment duration. The study was observational and cross-sectional; consequently, it cannot prove causation but demonstrate a statistical association.

## 5. Conclusions

Diabetic cardiac patients presented significant differences in the 25(OH)D level between the groups with respect to the degree of coronary atherosclerosis. A group of cardiac patients with diabetes with significant stenosis in three coronary arteries, hospitalized due to acute coronary syndrome, with the history of previous myocardial infarction and hyperlipidemia presented the lowest vitamin D level.

## Figures and Tables

**Table 1:** Characteristics of the examined group divided according to degree of coronary atherosclerosis (CASSS) into four subgroups.

	CASSS 0	CASSS 1	CASSS 2	CASSS 3	*p*
*N* and %	66 (20%)	79 (23%)	98 (29%)	94 (28%)	—
Age (years)^∗^	66.7 ± 8.0	68.8 ± 10.8	67.0 ± 9.9	69.8 ± 11.4	0.15
Sex (♀/♂)	33 (10%)/33 (10%)	30 (9%)/49 (14%)	26 (8%)/72 (21%)	30 (9%)/64 (19%)	<0.05
25(OH)D (ng/ml)^∗∗^	17.8 (6.4–43.9)	14.1 (5.4–38.2)	14.0 (5.9–36.7)	11.5 (4.0–28.6)	<0.001
Patients in groups according to the level of 25(OH)D					
0 to<10	8 (2%)	17 (5%)	15 (4%)	38 (11%)	<0.001
≥10 to <20	30 (9%)	40 (11%)	62 (19%)	36 (11%)	
≥20 to <30	23 (7%)	17 (5%)	15 (4%)	20 (6%)	
≥30	5 (2%)	5 (2%)	6 (2%)	0 (0%)	
BMI^∗∗^	30.6 (23.5–47.4)	30.1 (16.9–47.8)	28.7 (18.7–45.9)	29.0 (16.1–45.9)	0.19
Smoking (no/yes/ex-smokers)	47 (14%)/14 (4%)/5 (2%)	48 (14%)/24 (7%)/7 (2%)	63 (18%)/26 (8%)/9 (3%)	66 (20%)/20 (6%)/8 (2%)	0.82
Hypertension (no/yes)	5 (2%)/61 (18%)	9 (3%)/70 (20%)	6 (2%)/92 (27%)	5 (2%)/89 (26%)	0.45
Hyperlipidemia (no/yes)	27 (8%)/35 (12%)	25 (8%)/47 (15%)	46 (14.5%)/46 (14.5%)	40 (13%)/46 (15%)	0.25
CAD status (stable CAD/ACS)	58 (18%)/8 (2%)	42 (13%)/37 (10%)	57 (17%)/41 (12%)	49 (15%)/45 (13%)	<0.001
Previous MI (no/yes)	57 (17%)/9 (3%)	50 (15%)/29 (8%)	37 (10%)/61 (19%)	41 (12%)/53 (16%)	<0.001
Examination data (May–October/November–April)	16 (5%)/50 (15%)	14 (4%)/65 (19%)	22 (7%)/76 (22%)	18 (6%)/76 (22%)	0.74

^∗^Data presented as mean ± SD. ^∗∗^Data presented as median and range. Other data presented as the number of subjects (% of the whole group).

**Table 2:** Comparison between diabetic cardiac patients with and without significant stenosis.

	No significant stenosis (CASSS 0)	Stenosis (CASSS 1–3)	*p*
*N* and %	66 (20%)	271 (80%)	—
Age (years)^∗^	66.7 ± 8.0	68.5 ± 10.7	0.21
Sex (♀/♂)	33 (10%)/33 (10%)	85 (25%)/186 (55%)	<0.01
25(OH)D (ng/ml)^∗∗^	17.8 (6.4–43.9)	13.8 (4.0–38.2)	<0.001
Patients in groups according to the level of 25(OH)D			
0 to <10	8 (2%)	70 (21%)	<0.05
≥10 to<20	30 (9%)	138 (41%)	
≥20 to<30	23 (7%)	52 (15%)	
≥30	5 (2%)	11 (3%)	
BMI^∗∗^	30.6 (23.5–47.4)	29.2 (16.1–47.8)	0.09
Smoking (no/yes/ex-smokers)	47 (14%)/14 (4%)/5 (2%)	177 (52%)/70 (21%)/24 (7%)	0.51
Hypertension (no/yes)	5 (2%)/61 (18%)	20 (6%)/251 (74%)	0.93
Hyperlipidemia (no/yes)	27 (8%)/35 (12%)	111 (33%)/139 (47%)	0.99
CAD status (stable CAD/ACS)	58 (18%)/8 (2%)	148 (44%)/123 (36%)	<0.001
Previous MI (no/yes)	57 (17%)/9 (3%)	128 (38%)/144 (42%)	<0.001
Examination data (May–October/November–April)	16 (5%)/50 (15%)	54 (16%)/217 (64%)	0.61

^∗^Data presented as mean ± SD. ^∗∗^Data presented as median and range. Other data presented as the number of subjects (% of the whole group).

**Table 3:** Results of the Poisson regression analysis considering 25(OH)D level, age, sex, BMI, smoking habits, hypertension, hyperlipidemia, CAD status, previous MI, and examination data as determinants of CASSS.

	Determinants	OR	95% CI	*p*
CASSS	Age	0.005	−0.005 to 0.015	0.30
	Sex (♀/♂)	−0.101	−0.202 to −0.001	<0.05
	25(OH)D	−0.022	−0.036 to −0.007	<0.01
	BMI	−0.006	−0.024 to 0.013	0.54
	Smoking (no/yes/ex-smokers)	−0.014	−0.162 to 0.134	0.85
	Hypertension (no/yes)	−0.088	−0.278 to 0.102	0.36
	Hyperlipidemia (no/yes)	0.036	−0.059 to 0.130	0.46
	CAD status (stable CAD/ACS)	−0.059	−0.159 to 0.040	0.24
	Previous MI (no/yes)	−0.163	−0.262 to −0.064	<0.01
	Examination data (May–October/November–April)	0.012	−0.105 to 0.129	0.84

**Table 4:** Comparison of the selected parameters between the patients with stable CAD and ACS.

	Stable CAD	ACS	*p*
*N* and %	206 (61%)	131 (39%)	—
Age (years)^∗^	67.7 ± 10.1	68.9 ± 10.3	0.29
Sex (♀/♂)	69 (20%)/137 (41%)	50 (15%)/81 (24%)	0.37
25(OH)D (ng/ml)^∗∗^	16.1 (4.0–37.7)	12.5 (4.0–43.9)	<0.01
Patients in groups according to the level of 25(OH)D			
0 to <10	36 (11%)	42 (13%)	<0.05
≥10 to <20	106 (31%)	62 (18%)	
≥20 to <30	52 (15%)	23 (7%)	
≥30	12 (4%)	4 (1%)	
Patients in groups according to CASSS			
0	58 (17%)	8 (2%)	<0.001
1	42 (12%)	37 (11%)	
2	57 (17%)	41 (13%)	
3	49 (15%)	45 (13%)	
BMI^∗∗^	29.4 (16.1–47.4)	29.4 (16.9–47.8)	0.45
Smoking (no/yes/ex-smokers)	139 (41%)/42 (13%)/24 (7%)	84 (25%)/42 (12%)/5 (2%)	<0.01
Hypertension (no/yes)	18 (5%)/187 (56%)	7 (2%)/124 (37%)	0.96
Hyperlipidemia (no/yes)	84 (25%)/102 (30%)	54 (16%)/71 (21%)	0.73
Previous MI (no/yes)	149 (44%)/57 (17%)	37 (11%)/94 (28%)	<0.001
Examination data (May–October/November–April)	54 (16%)/151 (45%)	16 (5%)/115 (34%)	<0.05

^∗^Data presented as mean ± SD. ^∗∗^Data presented as median and range. Other data presented as the number of subjects (% of the whole group).

**Table 5:** Results of the logistic regression analysis considering 25(OH)D level, age, sex, BMI, smoking habits, hypertension, hyperlipidemia, and examination data as determinants of CAD status.

		OR	95% CI	*p*
CAD status	Age	−0.007	−0.036 to 0.022	0.63
	Sex (♀/♂)	0.293	−0.267 to 0.853	0.30
	25(OH)D	0.011	−0.028 to 0.049	0.58
	BMI	−0.004	−0.057 to 0.049	0.89
	Smoking (no/yes/ex-smokers)	−0.062	−0.495 to 0.372	0.78
	Hypertension (no/yes)	−0.081	−1.128 to 0.967	0.88
	Hyperlipidemia (no/yes)	−0.127	−0.677 to 0.424	0.65
	Previous MI (no/yes)	−1.685	−2.221 to −1.148	<0.001
	Examination data (May–October/November–April)	−0.844	−1.550 to −0.139	<0.05

**Table 6:** Results of the multiple regression analysis considering selected variables as determinants of 25(OH)D.

	Parameters of multiple regression analysis					
	Determinants	*β*	*p*	Multiple R2	F test	*p*
25(OH)D	Age	0.06	0.34	0.08	3.12	<0.01
	Sex (♀/♂)	0.02	0.73			
	BMI	0.08	0.17			
	Smoking (no/yes/ex-smokers)	−0.01	0.87			
	Hypertension (no/yes)	0.01	0.90			
	Hyperlipidemia (no/yes)	−0.13	<0.05			
	Previous MI (no/yes)	−0.13	<0.05			
	Examination data (May–October/November–April)	−0.19	<0.001			

Variable 25(OH)D was log transformed for this analysis.
